# Effect of Block Copolymer Self-Assembly on Phase Separation in Photopolymerizable Epoxy Blends

**DOI:** 10.1021/acs.macromol.4c00192

**Published:** 2024-05-15

**Authors:** Tanner L. Grover, C. Allan Guymon

**Affiliations:** Department of Chemical and Biochemical Engineering, University of Iowa, 4133 Seamans Center, Iowa City, Iowa 52242, United States

## Abstract

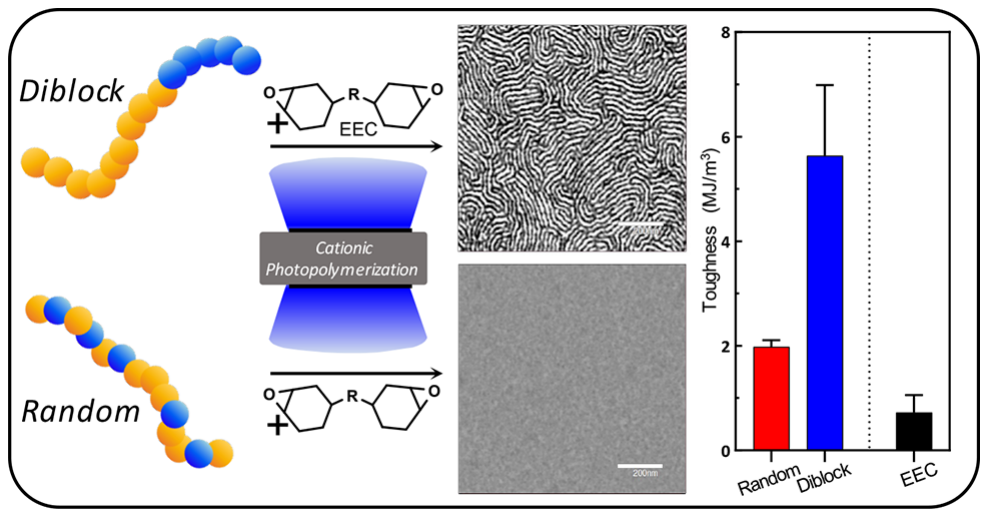

Directing self-assembly of photopolymerizable systems is advantageous for controlling polymer nanostructure and material properties, but developing techniques for inducing ordered structure remains challenging. In this work, well-defined diblock or random copolymers were incorporated into cationic photopolymerizable epoxy systems to investigate the impact of copolymer architecture on self-assembly and phase separated nanostructures. Copolymers consisting of poly(hydroxyethyl acrylate)*-x-*(butyl acrylate) were prepared using photoiniferter polymerization to control functional group placement and molecular weight/polydispersity. Prepolymer configuration and concentration induced distinctly different effects on the resin flow and photopolymerization kinetics. The diblock copolymer self-assembled into nanostructured phases within the resin matrix, whereas the random copolymer formed an isotropic mixture. Rapid photopolymerization and ambient temperature conditions during cure facilitated retention of the self-assembled phases, leading to considerably different composite morphology and thermomechanical behavior. Increased loading of the diblock copolymer induced long-range ordered cocontinuous structures. Even with nearly identical prepolymer composition, controlled nanophase separation resulted in significantly enhanced tensile properties relative to those of the isotropic system. This work demonstrates that controlling phase separation with a block copolymer architecture allows access to nanostructured photopolymers with unique and enhanced properties.

## 1 Introduction

Due to its insensitivity to oxygen, low polymerization shrinkage, and exceptional adhesion, cationic polymerization of epoxy resins has found considerable use in photopolymerization processes.^1−5^ However, the highly cross-linked and amorphous structure in these systems inhibits chain flow/deformation leading to low impact strength.^6−8^ This brittle mechanical behavior is even more pronounced as the cationic chain-growth polymerization limits stress dissipation during network formation causing high levels of internal stress.^9,10^ Alternatively, utilizing thermoplastics as miscible additives in thermally initiated epoxy resins has shown promise in addressing similar mechanical property deficiencies.^11−13^ Blending rubbery (low glass transition temperature, *T*_g_) thermoplastics with cross-linking epoxy systems can produce dual phase morphology through polymerization-induced phase separation (PIPS) with localized soft domains embedded in the cross-linked matrix.^14,15^ The soft domains dispersed within the thermoset network plastically deform and cavitate under stress, thereby preserving structural integrity and extending fracture resistance.^16^ The morphology of modified epoxies is established by the architecture and concentration of the thermoplastic, which dictates physicochemical interactions in the resin matrix before and during cure. Therefore, the physical and chemical nature (i.e., miscibility, reactivity, and molecular weight) of the additive is an essential consideration when blending these components.^8,11^ Importantly, block copolymers (BCPs) prepared through controlled polymerization techniques can form ordered nanostructures by self-assembling in thermosetting resins.^17−22^ The covalent bonding between blocks enables a high interfacial connectivity between phases and enhances material toughness. Despite advances in thermally cured epoxies, much less attention has been given to thermoplastic enhancement and self-assembly of photocurable systems.^23−25^ Therefore, precise regulation of the BCP architecture and investigating the additive influence on phase-separated morphologies may facilitate enhanced properties in photopolymerized materials.

Of particular interest are amphiphilic BCPs containing chain segments with immiscible chemistries that may offer control over self-assembly and phase separation in photocurable systems by acting as macromolecular directors. Utilizing BCP preparations that allow high-level control over architecture is crucial to tailor BCP/matrix interactions and individual phase properties and to minimize or eliminate macroscopic phase separation.^26^ Photoiniferter polymerization is a controlled radical polymerization (CRP) technique that offers exceptional control over functional group placement, molecular weight, dispersity, and block volume fraction.^27,28^ The photoiniferter process typically employs trithiocarbonate species as a photoinitiator, chain transfer, and a reversible termination agent to facilitate controlled polymerization. Since there is no need for an exogenous initiator, concentration of irreversibly terminated chains is considerably reduced, permitting facile block addition with subsequent monomer feeds.^29,30^ Additionally, the polymerization can be conducted at room temperature in a closed system which reduces iniferter degradation and monomer evaporation, thereby allowing molecular weight to be targeted more accurately relative to thermally initiated CRP systems, as shown in our recent work.^31^ Ultimately, this process enables precise control over polymer architecture, which may facilitate targeted interactions within a photocurable matrix.

The utility of the regulated copolymer architecture for controlling morphology in photopolymerizable systems has been demonstrated elsewhere. For example, Scholte et al. used nitroxide-mediated polymerization to selectively place epoxy-functionalized repeat groups as the end segments of a poly(butyl acrylate) gradient triblock copolymer which subsequently induced microphase separation when combined with a photopolymerizable epoxy system.^32^ One phase consisted of the epoxy-functionalized segment covalently bonded with the epoxy network while the other phase was rich in the nonreactive BCP segment. The combination of highly cross-linked and deformable domains significantly increased toughness of the photocured material. Moreover, Naguib et al. used atom transfer radical polymerization to manipulate nonreactive block length in a diblock copolymer consisting of poly(ethylene oxide)*-block-*(glycidyl methacrylate), inducing considerable increases in epoxy fracture energy with a larger nonreactive block owing to greater network mobility.^33^ A more recent study applied radical addition/fragmentation polymerization to vary the molecular weight of triblock copolymers consisting of a highly amphiphilic poly(hydroxyethyl acrylate)*-grad-*(butyl acrylate)*-grad-*(hydroxyethyl acrylate) for controlling morphology in a free-radical photopolymerizable system through hydrophobic/hydrophilic interactions.^34^ The gradient amphiphile produced soft and hard domains with size scales dependent on the copolymer molecular weight. The dual phase behavior along with increased hydrogen bonding from pendant hydroxyl groups led to greater elongation and toughness relative to that of the unfilled system. However, it was not evident whether the resulting network structure was a result of gradient BCP self-assembly or PIPS.

Hydroxyl-functionalized copolymers have been incorporated as additives in photocurable epoxy resins due to the potentially beneficial epoxy/alcohol reaction in cationic polymerization.^31,35^ The chain transfer mechanism provides covalent bonding between the additive and network matrix reinforcing domain adhesion.^36^ The presence of hydroxyl pendant groups on branched copolymers increases cationic photopolymerization rate and conversion while also increasing *T*_g_ of the epoxy polymer in grafted blends.^37^ Furthermore, relative to an epoxy pendant group, hydroxyl groups are considerably more polar. Therefore, coupled with diblock architecture, utilizing hydroxyl pendant groups as the reactive functionality on BCPs could enable greater amphiphilic character, which may lead to greater self-assembly potential and control over phase-separated morphology.

In this work, we examined the effects of the BCP hydroxyl group placement on self-assembly, phase morphology, and mechanical properties in a photocurable epoxy system. Photoiniferter polymerization was used to prepare amphiphilic diblock and random poly(hydroxyethyl acrylate)-*x*-poly(butyl acrylate) with similar molecular weight and chemical composition as determined by NMR and gel-permeation chromatography. Copolymers were incorporated into a photocurable epoxy resin over a range of concentrations. The impact of copolymer inclusion and self-assembly on resin flow properties before and during photocuring was analyzed via rheometry. Since self-assembly can alter photopolymerization kinetics through reactive group segregation, photodifferential scanning calorimetry was used to compare the instantaneous polymerization rates between diblock and random copolymer formulations. In the cured state, dynamic mechanical analysis was used to relate thermomechanical behavior to the polymer phase structure. The nanostructure and phase-separated morphology were examined spatially by using atomic force microscopy. The presence of self-assembly and characteristic phase structure was confirmed using X-ray scattering before and after photocuring. Finally, mechanical testing was used to determine the effect of controlled phase separation and structure on bulk material properties. We hypothesized that utilizing the self-assembly of a diblock copolymer could enable control over nanostructure in photocurable materials, leading to enhanced material properties. By holding chemical composition constant and controlling functional group placement, we have thereby developed a greater understanding of the role of BCP architecture on polymer blend phase morphology and properties.

## 2 Materials and Methods

### 2.1 Materials

#### Copolymer Synthesis and Characterization

**Figure 1:**
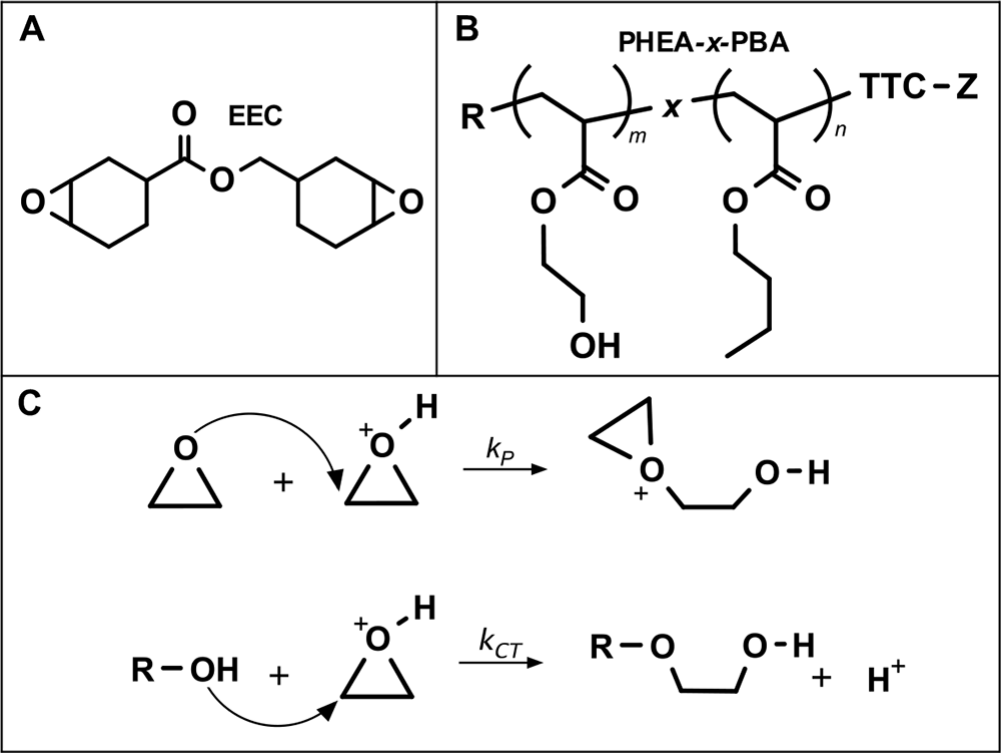
(A) Structure of diepoxide cross-linker, EEC, and (B) PBA-*x*-PHEA block or random copolymer. (C) Propagation (top) and chain transfer (bottom) mechanisms of epoxy cationic polymerization in the presence of hydroxyl functional groups.

Hydroxyethyl acrylate (HEA), butyl acrylate (BA), and 2-(dodecylthiocarbonothioylthio)-2-methylpropionic acid (DDMAT) were purchased from Sigma-Aldrich and used without further purification for copolymer synthesis. Block copolymers were prepared using photoiniferter polymerization, where DDMAT was used as the photoinitiator, chain-transfer, and reversible termination species. The molar composition needed to achieve a target polymer molecular weight was determined using eq 1:^31,38^
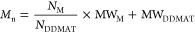
1where *M*_n_ is the number-average polymer molecular weight, *N*_M_/*N*_DDMAT_ is the mole ratio of monomer to photoiniferter, and MW_M_ and MW_DDMAT_ are the molecular weights of monomer and photoiniferter, respectively. The diblock copolymer (PHEA*-b-*PBA, Figure 1B) was prepared by combining HEA and DDMAT at a mole ratio of 30:1 in a 250 mL Schlenk flask followed by diluting to 50 wt % in THF. Ambient oxygen was removed from the reaction solution by three freeze–pump–thaw cycles and backfilled with an argon atmosphere. The bottom of the Schlenk flask was placed 1.5 cm away from a 445 nm LED to give a light intensity of 15 mW/cm^2^ at the bottom of the solution surface. The mixture was exposed for 24 h to achieve a monomer conversion of >95% as confirmed by disappearance of the C=C stretching peak at 1637 cm^–1^ using infrared spectroscopy (Nexus 670, Thermo Nicolet). A second monomer feed of BA diluted to 75 wt % in THF was charged to the reaction flask at a mole ratio of 200:1 of BA to PHEA-DDMAT. The reaction was once again evacuated of air using three freeze–pump–thaw cycles and backfilled with argon. The reaction solution was exposed for another 24 h to achieve >95% monomer conversion. The random copolymer (PHEA*-co-*PBA, Figure 1B) was prepared by combining HEA, BA, and DDMAT at the same overall mole ratios as the diblock (200:30:1 HEA:BA:DDMAT) and diluted to 75 wt % in THF. Similarly, the random copolymer reaction solution was exposed until >95% monomer conversion was achieved. THF and residual monomer were removed using rotary evaporation and subsequent drying under vacuum at ambient temperature overnight.

Copolymer number-average molecular weight, *M*_n_, and molecular weight distribution, *M*_w_/*M*_n_, were determined by using gel permeation chromatography (GPC, column: PLgel Mixed-D, Agilent; mobile phase: THF, 1.0 mL/min; detector: differential refractive index, Shimadzu). The column was calibrated using polystyrene standards ranging from 4 to 160 kDa (*Đ* = 1.02–1.04). The mole percent of hydroxyl functional groups in each copolymer was determined using ^1^H NMR (CDCl_3_, AV-500 MHz, Bruker) by comparing the peak area of ester methylene protons (δ = 4.1 ppm) of the BA pendant group to that of the hydroxyl methylene protons (δ = 3.8 ppm) of the HEA pendant. Copolymers were diluted to 50 wt % in acetone and stored in the dark at −15 °C. These solutions allowed rapid dissolution of copolymers in subsequent photopolymerizable formulations.

#### Sample Preparation and Photopolymerization

The photocurable epoxy resin consisted of 3,4-epoxycyclohexylmethyl 3,4-epoxycyclohexanecarboxylate (EEC, Sigma) as a diepoxide cross-linker (Figure 1A), triarylsulfonium hexafluoroantimonate salts (2 wt %, TSA, Sigma) as a photoinitiator, and isopropyl thioxanthone (1 wt %, ITX, Irgacure) as a photosensitizer. All chemicals were used as received. Diblock and random copolymer solutions were combined with epoxy resin, and acetone was removed using rotary evaporation to yield bulk formulations. Polymer films were prepared by pipetting formulations onto RainX-polished glass molds separated a set distance using 0.1 mm spacers. Samples were then exposed to a 405 nm light (LED Spot 100 IC, Honle) at an incident intensity of 15 mW/cm^2^ for 5 min to initiate cationic photopolymerization. During polymerization, active cationic centers can propagate through hompolymerization of epoxy or participate in chain transfer with hydroxyl groups as shown in Figure 1C. After photocuring, samples were thermally annealed at 115 °C for 1 h to achieve full conversion of epoxy groups. Conversion was determined by disappearance of the oxirane stretching band bracketing 788 cm^–1^ using infrared spectroscopy, as shown in the Supporting Information (Figure S1).^39^ All cured samples were analyzed after thermal annealing, unless noted otherwise.

### 2.2 Methods

The dynamic viscosity and photopolymerization gel point were measured by using a rheometer (Kinexus Ultra Plus, Netschz) with parallel plate geometry. Uncured samples were placed on a glass stage and sandwiched to a gap height of 100 μm using the upper plate (8 mm diameter, stainless steel). An oscillation amplitude of 1 Hz with 1% shear strain was applied to measure the rheologic properties before and during photocuring. Samples were irradiated through the transparent glass stage using a 410 nm LED with an output of 20 mW/cm^2^ at the bottom sample surface while measuring the storage and loss modulus of the material. Here, the polymerization gel point is defined at the time the storage modulus exceeds the loss modulus.

Photodifferential scanning calorimetry (P-DSC, Pyris 1, PerkinElmer) was used to monitor heat evolution during photopolymerization. The P-DSC was fitted with a 405 nm light (LED Spot 100 IC, Honle) to give an irradiation intensity of 15 mW/cm^2^ at the sample surface. Samples weighing 1.75 ± 0.25 mg were placed into crimped aluminum crucibles and loaded into the P-DSC sample cell. Sample and reference cells were covered with a hood containing a quartz window that allowed light transmission and maintained an inert N_2_ atmosphere. The instantaneous polymerization rate was calculated using eq 2:
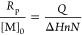
2where *R*_p_/[M]_0_ is the normalized polymerization rate, *Q* is the measured heat flow, Δ*H* is the heat of polymerization per mole of epoxy functional groups (114 kJ/mol),^40^*n* is the number of epoxy groups per monomer, and *N* is the moles of monomer in the sample.

Thermomechanical relaxations of cured films were examined by using a dynamic mechanical analyzer (DMA Q800, TA Instruments). DMA samples consisted of photopolymerized films cut into strips with rectangular dimensions of 12 × 2 × 0.1 mm^3^ (*L*·*W*·*D*). The loss factor (tan δ) was measured using DMA with a frequency of 1 Hz and an oscillation amplitude of 15 μm while ramping the temperature from −70 to 300 °C over two cycles. The second heating cycle is reported. For this work, maxima of the tan δ versus temperature curves are considered as the domain glass transition temperature.^32,41^

Atomic force microscopy (AFM, MFP-3D, Asylum Research) in tapping mode was used to determine nanostructure and phase morphology of photocured thin films. 1 μm × 1 μm phase images were obtained by scanning an oscillating nanosized probe (SPM Probe, 0.3–0.8 N/m force constant, MikroMasch) across the photopolymerized film surface at a tapping rate of 1 Hz with an image set point of 1.2 V. The mechanical and adhesive interaction between the probe and material is measured by a change in oscillation/phase angle. Output data were processed using Igor software (WaveMetrics).

Ordered structure on the nanoscale was investigated using small-angle X-ray scattering (SAXS, Xuess 2.0, Xenocs). Samples were examined before and after photopolymerization and additionally after thermal annealing. Prephotopolymerized samples were loaded into a cylindrical sample cell (4 mm in length) using adhesive Kapton films to seal the ends. Photocured and annealed films were removed from the glass molds and placed directly within the beam path. A copper X-ray source was used, and the sample-to-detector distance was 2546.3 mm. Scattering data were acquired for 10 min and processed using Foxtrot software.

Photocured film tensile properties were determined using the DMA Q800 in controlled force mode at room temperature. For stress–strain analysis, cured thin films were clamped in a DMA tensile fixture and subjected to an increasing pressure load at a constant force rate of 1 N/min until material failure. The tensile modulus was determined using the initial slope (<0.1% strain) of the stress/strain curve. The ultimate tensile strength and elongation are noted at the point of material failure, and toughness was calculated by integrating the stress/strain curve. Additionally, creep testing was used to examine deformation behavior under constant load and after load removal. Tensile bars were subjected to an instantaneous and constant pressure of 5 MPa for 10 min and then released. Material strain was continuously monitored as a function of time.

## 3 Results and Discussion

Photoiniferter polymerization provides excellent control over the polymer molecular weight and molecular weight distribution. Because of the reversibly terminated chain ends, subsequent monomer feeds can be used to modify polymers with additional functionality. The diblock copolymer for this work was prepared by initially polymerizing HEA to yield a PHEA homopolymer capped with the initiating chain transfer agent (Macro-CTA). A second monomer feed consisting of the BA monomer was combined with the Macro-CTA to polymerize the second block, yielding the “Diblock” amphiphilic copolymer. Because of the amphiphilicity and low polydispersity of the copolymer,^42^ it was hypothesized that these immiscible chemistries could drive controlled nanophase separated structures in solution. Alternatively, copolymerization of HEA and BA resulted in the “Random” copolymer with hydroxyethyl and butyl pendants randomly substituted along the copolymer chain. The random polymerization incorporated contrasting pendant groups along the entire copolymer such that the driving force for the self-assembly of chains was considerably reduced. Thus, an isotropic material would likely be formed when incorporated into a photocurable resin matrix. Ultimately, the precise control over both copolymer preparations allowed valid comparisons of varied architectures while holding the molecular weight, polydispersity, and chemical concentration approximately constant.

**Figure 2:**
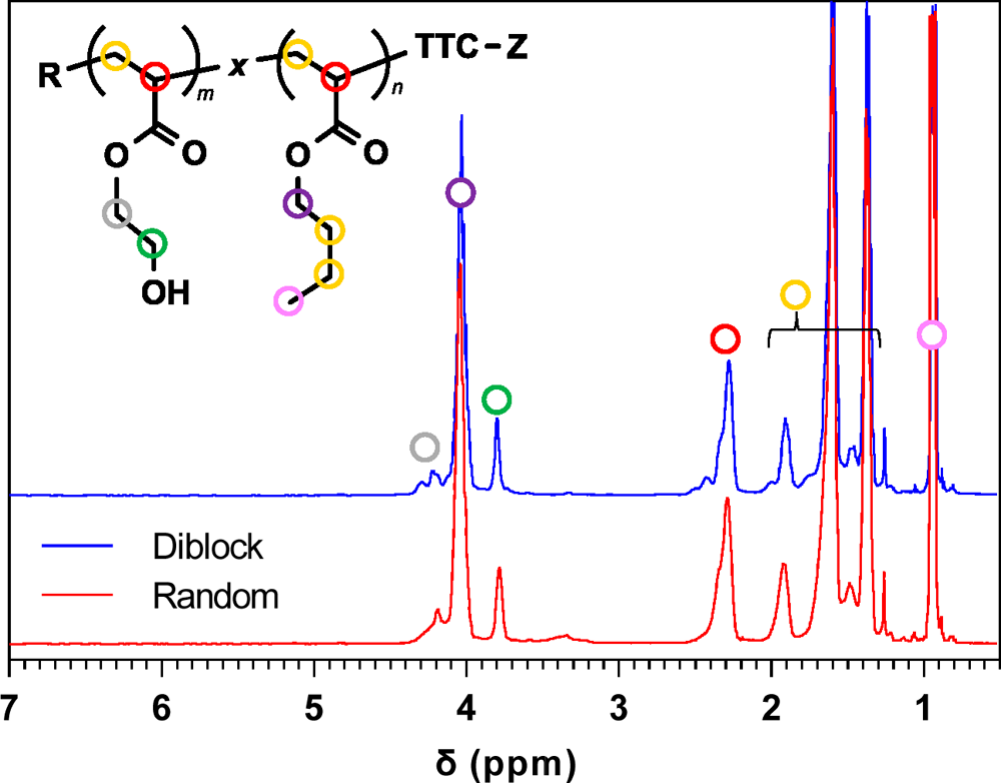
^1^H NMR spectra and peak assignment for PHEA*-b-*PBA ('Diblock') and PHEA*-co-*BA ('Random') copolymers.

Figure 2 and Table 1 show the H NMR and GPC characterizations for these two copolymers. The NMR peak assignment and relative peak areas showed that the chemical compositions of copolymers were very similar. For example, the mole percentage of pendant hydroxyl groups was determined to be 14.7 and 14.3 for the diblock and random copolymers, respectively. Moreover, the number-average molecular weight determined using GPC for both polymers was very similar to only a 4% difference. Ultimately, these findings indicated that any effects of BCP architecture on epoxy composite structure should be a strong function of pendant group placement and should be nearly independent of initial chemical composition.

**Table 1:** Tabulated Copolymer Number-Average Molecular Weight, Polydispersity, and Chemical Composition

polymer	structure	*M*_n_^a^ (kg/mol)	*M*_w_/*M*_n_^a^	mol % HEA^b^
macro-CTA	PHEA	3.4	1.07	100
Diblock	PHEA*-*b*-*PBA	25.4	1.12	14.7
Random	PHEA*-*co*-*PBA	26.5	1.12	14.3

a Determined using gel permeation chromatography.

b Mole percentage of hydroxyl groups per copolymer determined using ^1^H NMR and peak integration

The diblock and random copolymers were combined with the photocurable epoxy resin at three concentrations. It should be noted that incorporating either copolymer in the epoxy resin matrix did not result in any visible phase separation (e.g., opacity or cloudiness) at any concentration examined. The lack of macroscopic phase separation suggested that these network modifiers were well dispersed in this resin. However, the distinctly different copolymer architectures could display different flow behaviors in the uncured epoxy matrix due to intermolecular interaction differences. More specifically, the segregation of polar and nonpolar segments along the diblock copolymer could have different impacts on resin viscosity compared to the randomly substituted system.

**Figure 3:**
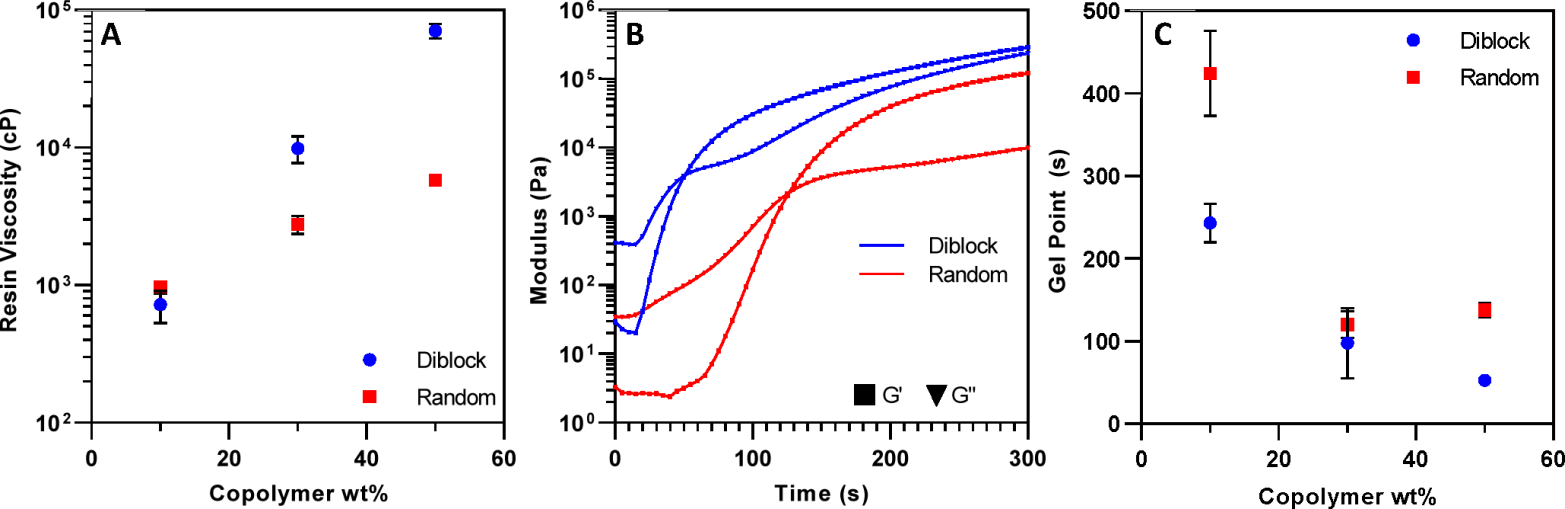
(A) Photocurable epoxy resin dynamic viscosity as a function of copolymer architecture and concentration. (B) Representative rheological property evolutions during photopolymerization of 50 wt % random and diblock formulations. The point at which the storage modulus, *G*′, intersects the loss modulus, *G*″, is defined in this work as the reaction gel point. (C) Reaction gel point as a function of copolymer concentration for the random and diblock copolymer epoxy systems. Photopolymerization was conducted using a 410 nm LED, with 20 mW/cm^2^ at the resin surface. Error bars represent the standard deviation of three replicates.

Figure 3A shows the dynamic viscosity of the uncured resin as a function of the copolymer structure and concentration. As would be expected, increasing the concentration of high-MW species increased the viscosity of the blends independent of architecture. This general increase in viscosity was most likely due to the greater interfacial attractions imparted by the increasing concentration of macromolecules in the system.^25^ However, when the two additives were compared at increasing concentrations, the diblock copolymer increased viscosity to a significantly greater degree than the random copolymer. For example, increasing the concentration of the diblock copolymer from 30 to 50 wt % increased the blend viscosity by nearly an order of magnitude, whereas this same increase in concentration for the random copolymer formulation resulted in only a 2-fold increase. It is possible that the greater segregation of contrasting chemistries on the diblock copolymer may have promoted greater interactions/entanglements between the hydrophobic segments.^43^ Additionally, the increased level of hydrogen bonding between hydroxyl segments and epoxy ester groups could also be responsible for the greater increase in viscosity. This segregation effect became more pronounced at higher concentrations with the 50 wt % diblock formulation displaying a 12-fold increase in viscosity relative to the random system. Ultimately, these increased intermolecular interactions in the more segregated diblock system inhibited chain flow, resulting in greater viscosity.

Similarly, the flow behavior of the material was examined during photopolymerization to understand the relationship between molecular interactions and polymer network structure evolution. In cationic homo-photopolymerizations, light exposure of the resin produces cations that propagate through an active chain-end mechanism to form the polymer network. Alternatively, the presence of hydroxyl groups changes this mechanism as the hydroxyl reacts with the protonated epoxy ring, displacing the active center through chain transfer and returning a hydroxyl group (Figure 1). This chain transfer mechanism enabled covalent bonding between the copolymer and epoxy network, forming graft structures in these composite systems (Figures S3 and S4A–C). Therefore, it was reasonable to believe that altering the copolymer morphology would have distinct effects on the rheological properties and kinetics of these systems during photopolymerization.

Photopolymerization gel point as a function of concentration for the diblock and random copolymer systems are shown in Figure 3C. Upon photoinitiation, the distinct juncture where the material behaved more as an elastic solid as opposed to a viscous liquid (e.g., *G*′ > *G*″, Figures 3B and S2A,B) was approximated as the gel point. At 10 wt %, a significant reduction in gel point was observed for the diblock relative to random, suggesting that copolymer architecture played a significant role in the polymerization process. Further increases in concentration reduced the gel point for both copolymer structures possibly due to increased resin viscosity. However, the diblock systems consistently displayed lower gel points with nearly a 2-fold reduction at the 50 wt % comparison. These rheology findings suggest that the diblock architecture possibly induced self-assembly of the resin matrix. This organization may have increased local concentrations of reactive groups, causing cross-linking and gelation to occur more rapidly.

**Figure 4:**
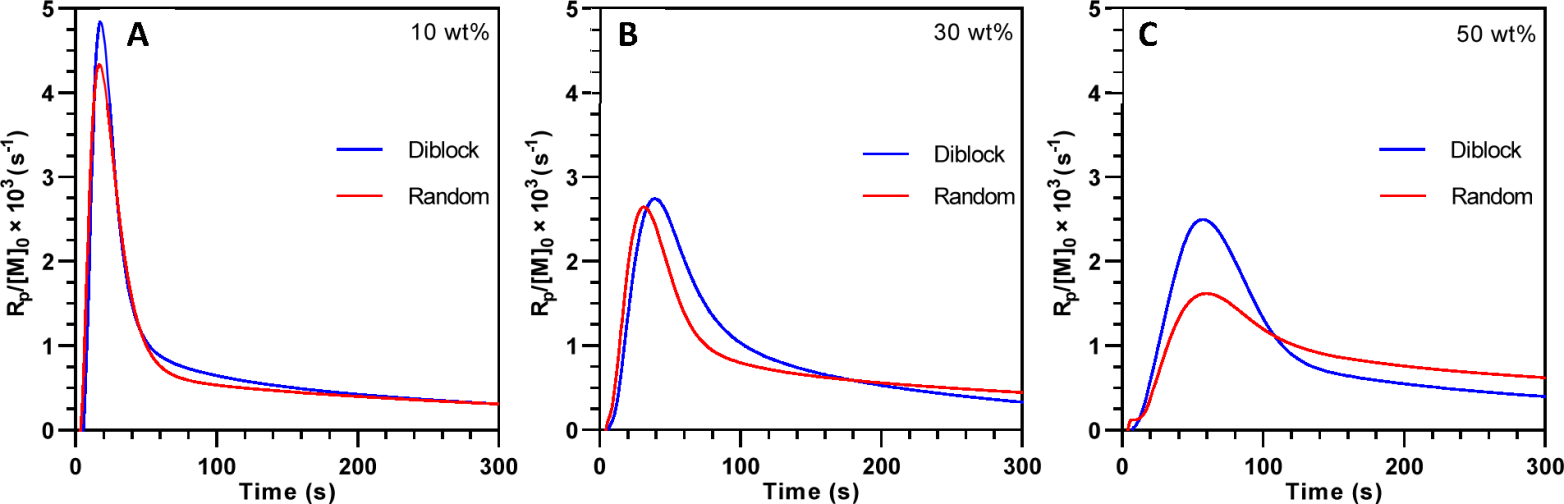
Representative normalized polymerization rate as a function of time and concentration for (A) 10 wt %, (B) 30 wt %, and (C) 50 wt % random or diblock copolymer modified epoxy systems. The rate was determined using P-DSC, and photopolymerization was initiated with a 405 nm LED at an intensity of 15 mW/cm^2^ at the sample surface.

As shown in other reports, chemical structure and organization of the prepolymer resin significantly influence photopolymerization kinetics.^44^ For example, prior work has shown that dispersed surfactants impart self-organization of the monomer matrix into lyotropic liquid crystal (LLCs) phases prior to photopolymerization.^45,46^ Depending on the physical interactions between species in solution, organization into hydrophobic and hydrophilic domains can be established, which stems from the amphiphilicity of the surfactant. This preorganization induces monomer segregation and increases local concentration of reactive groups and overall polymerization rate. To determine any changes in rate that would indicate system segregation, the polymerization rate was determined for these composite systems using P-DSC. Shown in Figure 4A–C is the normalized polymerization rate for diblock and random copolymer formulations at different concentrations. Regardless of copolymer modifier, the 10 wt % blends exhibited rapid onset of polymerization and relatively high maximum rate. Interestingly, even with similar rate behavior, the 10 wt % diblock system displayed a slightly higher maximum polymerization rate compared to the random formulation. Further increases in copolymer concentration to 30 wt % (Figure 4B) also showed a slightly higher maximum polymerization rate for the diblock-filled system. At 50 wt % (Figure 4C), the changes in photopolymerization kinetics became much more apparent. The diblock system showed a 50% increase in the maximum polymerization rate relative to the random formulation. The change in polymerization rate between copolymer structures with increasing concentration appears to be similar to principles from LLC systems in which increasing amphiphilic species concentration leads to enhanced segregation of reactive groups.^47^ Indeed, an increased concentration of the diblock copolymer should change miscibility in the uncured epoxy resin. The epoxy cross-linker, containing hydrogen bond acceptor sites, may have had greater attraction to the hydroxyl-substituted segment of the diblock copolymers, and as the concentration of copolymer was increased, the hydroxyl segment and epoxy phase would become less miscible with the hydrophobic copolymer segment. Therefore, greater segregation between phases may have increased the local concentration of reactive species, leading to faster polymerization rates.

**Figure 5:**
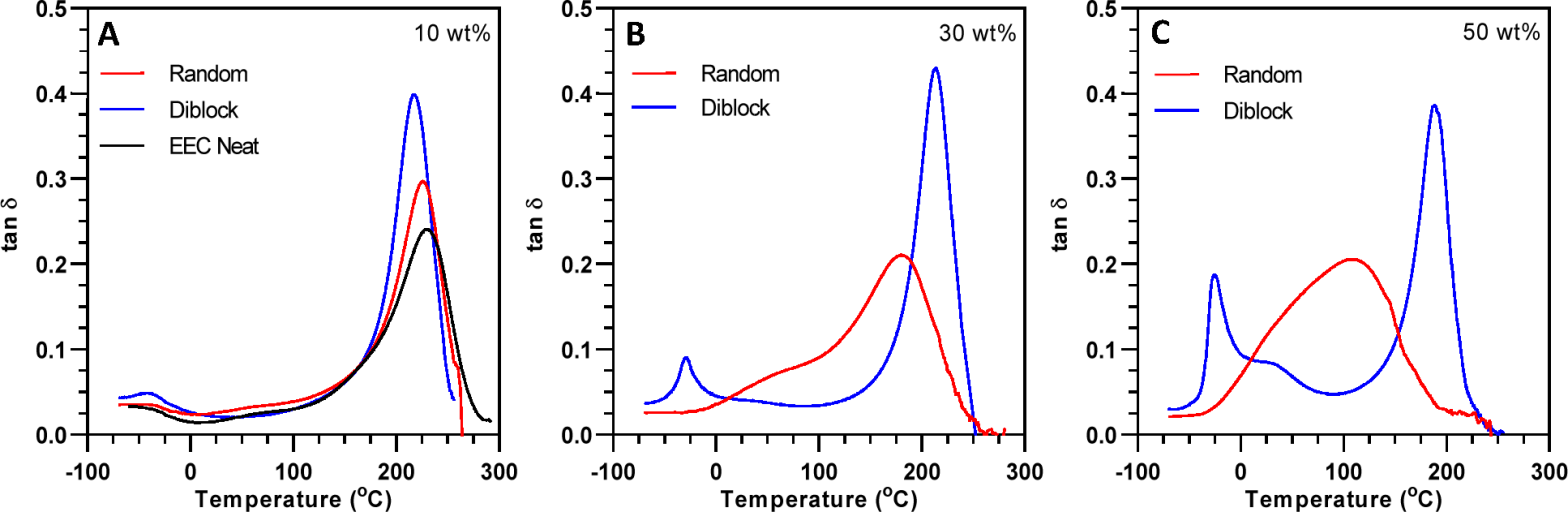
Tan δ as a function of temperature and concentration for (A) 10 wt %, (B) 30 wt %, and (C) 50 wt % of random and diblock copolymer-modified epoxy networks. Polymer films were fabricated by photocuring formulations for 5 min using a 405 nm LED at 15 mW/cm^2^ and then thermally postcuring at 115 °C for 1 h. Rectangular films were subjected to an oscillating tensile strain (1 Hz) at an amplitude of 15 μm while ramping the temperature. The second heating cycle is reported.

The indication of phase segregation occurring through rheology and kinetic analysis suggested that phase-separated structures may be incorporated into the cured material, which should be manifested through bulk material properties. Dynamic mechanical analysis was used to examine thermal relaxations over a broad temperature range. Depending on connectivity within individual phases, materials may display distinct thermomechanical properties (e.g., *T*_g_). Figures 5A–C show tan δ behavior as a function of temperature for the two copolymer-filled formulations at three concentrations. As a comparison, the tan δ for the unfilled epoxy material is included in Figure 5A with the maximum of the trace considered as the *T*_g_.^48^ The neat epoxy system displayed a maximum slightly above 200 °C, which was assigned to the *T*_g_ of the cross-linked network. At 10 wt %, incorporation of the random copolymer shifted the *T*_g_ of the bulk epoxy network approximately 5 °C lower but otherwise demonstrated relatively low impact on the thermomechanical behavior over this temperature range. The diblock copolymer at 10 wt % reduced the bulk *T*_g_ by −15 °C, which most likely stemmed from insertion and grafting from the more rubbery, hydroxyl-substituted copolymer segment into the epoxy network. Additionally, a small local maximum appeared at approximately −35 °C for the diblock formulation. This secondary transition was attributed to the *T*_g_ of a domain composed mainly of the nonreactive poly(butyl acrylate) copolymer segments. A pure poly(butyl acrylate) homopolymer has a reported *T*_g_ of −49 °C,^43^ so the higher *T*_g_ relative to the literature in this dual phase system possibly stemmed from the restricted mobility imparted by stiff cross-links in the adjacent phase and very light cross-linking occurring in this phase.

As the concentration of copolymer was increased to 30 wt %, the differences in composite structure and phase transitions became more prominent (Figure 5B). The random copolymer at 30 wt % reduced the network *T*_g_ to 180 °C while still exhibiting a single transition indicative of a single-phase, isotropic material. The large reduction in *T*_g_ was likely a consequence of the epoxy network chemically connecting at hydroxyl pendant groups along the entire copolymer length, and the soft/rubbery copolymer reducing cross-link density and network stiffness. Conversely, the diblock copolymer system maintained two thermomechanical transitions and bulk network *T*_g_ to a greater extent. The low-temperature transition became more prominent at this concentration due to a higher volume fraction of the block copolymer in the formulation. The hydroxyl–epoxy reactivity and effects on phase separation were further examined by synthesizing diblock copolymers with fewer hydroxyl pendant groups while holding MW constant. Reducing block fraction of HEA repeat units was shown to increase phase separation characteristics and considerably reduce mechanical integrity due to low connectivity between the copolymer and epoxy network (Figure S4A–C).

Further increasing concentration to 50 wt % (Figure 5C), the random BCP formulation displayed a single *T*_g_ at about 109 °C. The tan δ maximum for this formulation is approximately the average of transition temperatures for the phase-separated diblock system, showing greater integration of this copolymer in the epoxy network. On the other hand, the 50 wt % diblock formulation displayed more pronounced phase-separated characteristics by increasing the tan δ maximum of the lower temperature transition. For the bulk network, the *T*_g_ was reduced to 190 °C, relative to the 30 wt % formulation, which indicated an increased concentration of the hydroxyl-functionalized copolymer segment in the cross-linked epoxy network. Additionally, a shoulder at approximately 25 °C for the diblock system was apparent and proposed to be a consequence of cross-linking occurring in this phase due to residual HEA monomer being incorporated along the PBA segment during BCP synthesis. This hypothesis was tested by reversing BCP block addition, i.e., polymerizing BA first followed by chain extension with HEA. The 25 °C shoulder was absent for this EEC/BCP blend, suggesting this cross-linking mode was minimized, as shown in Figure S5.

**Figure 6:**
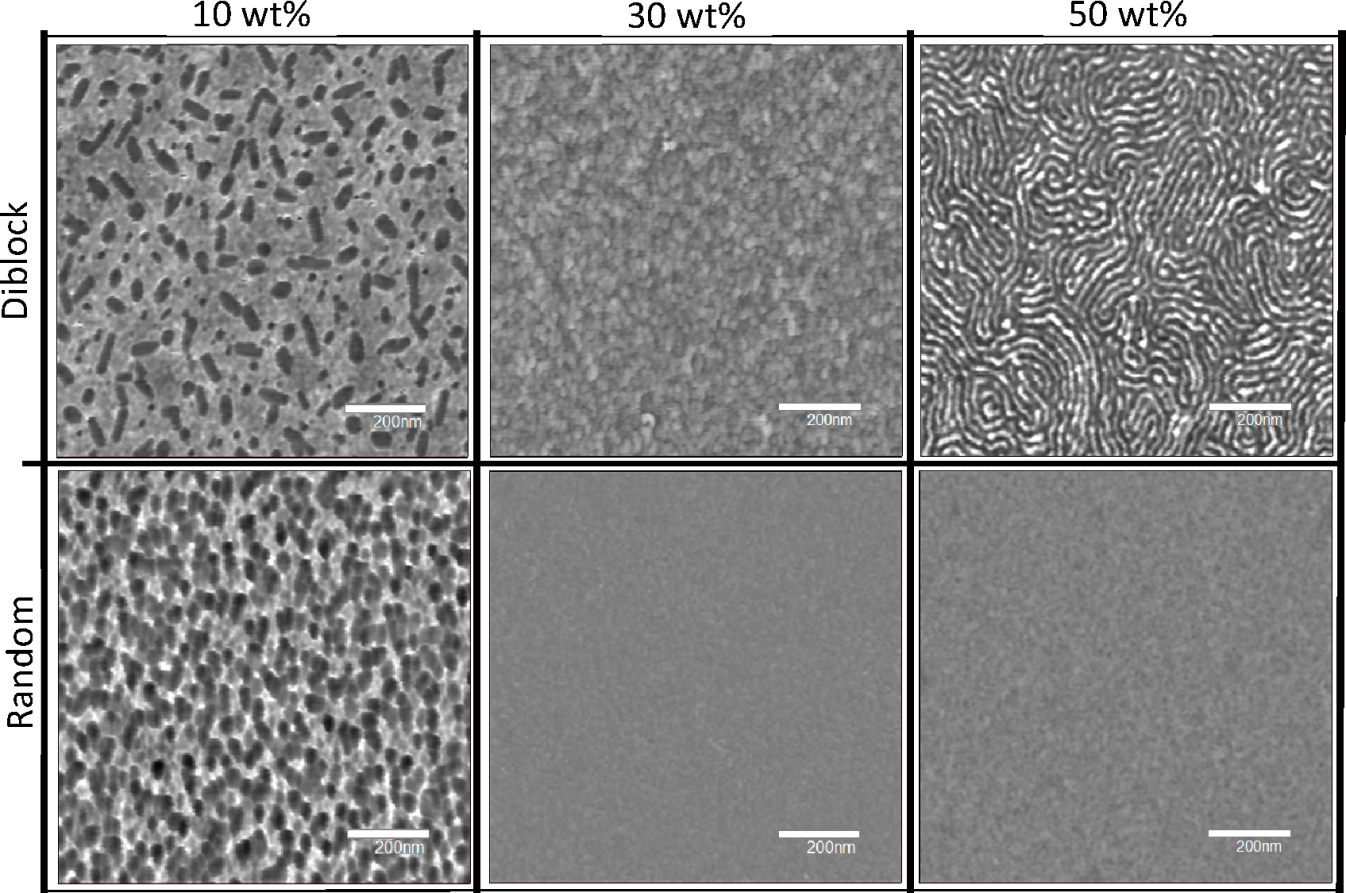
AFM phase images of epoxy films modified with either diblock (top row) or random (bottom row) copolymers at 10 wt % (left column), 30 wt % (middle column), and 50 wt % (right column). Samples were prepared by photocuring formulations (405 nm, 15 mW/cm^2^, 5 min) and subsequent thermal annealing (115 °C, 1 h). Images dimensions are 1 × 1 μm^2^, and the scale bar represents 200 nm. All images are normalized to a difference in the phase angle of ±15°.

To examine the polymer network structure and phase separation spatially, atomic force microscopy (AFM) phase imaging was used. The mechanical and adhesive interaction between the AFM probe and material allows for detection of nanoscale modulus differences across a polymer surface and is useful for characterizing the phase-separated network structure.^49^Figure 6 contains 1 × 1 μm^2^ AFM phase images for the copolymer-filled epoxy films. Spherical domains with sharp contrast across the domain interface were observed for the 10 wt % diblock formulation. The phase-separated structure on this size scale suggested that the hydrophobic segments form a lightly cross-linked (low *T*_g_) domain through physical entanglements with covalent bonding across the domain interface to the hydrophilic segment and epoxy network (high *T*_g_). The sharp contrast change across domain interfaces for the block system was not as readily apparent in the random formulations at the same concentration. This reduction in phase angle difference was likely due to greater integration of the random copolymer into the network by covalent bonding along the entire copolymer length. These formulations exhibited greater homogeneity in phase angle by increasing the random copolymer concentration, correlating with single-phase thermomechanical behavior as shown in the tan δ results. Interestingly, the diblock copolymer at 50 wt % prominently exhibited an ordered lamellar structure with relatively long-range, cocontinuous phases composed of high- and low-modulus domains, respectively, approximately 20 nm in width. At this concentration, the mass fraction of the hydrophobic copolymer segment in the system was much closer to the fraction of epoxy/hydrophilic segments which may have driven greater immiscibility between the two phases. Interestingly, this evidence of controlled nanophase separation and long-range order in a solvent-free system is unique among photopolymerized thermosets.

**Figure 7:**
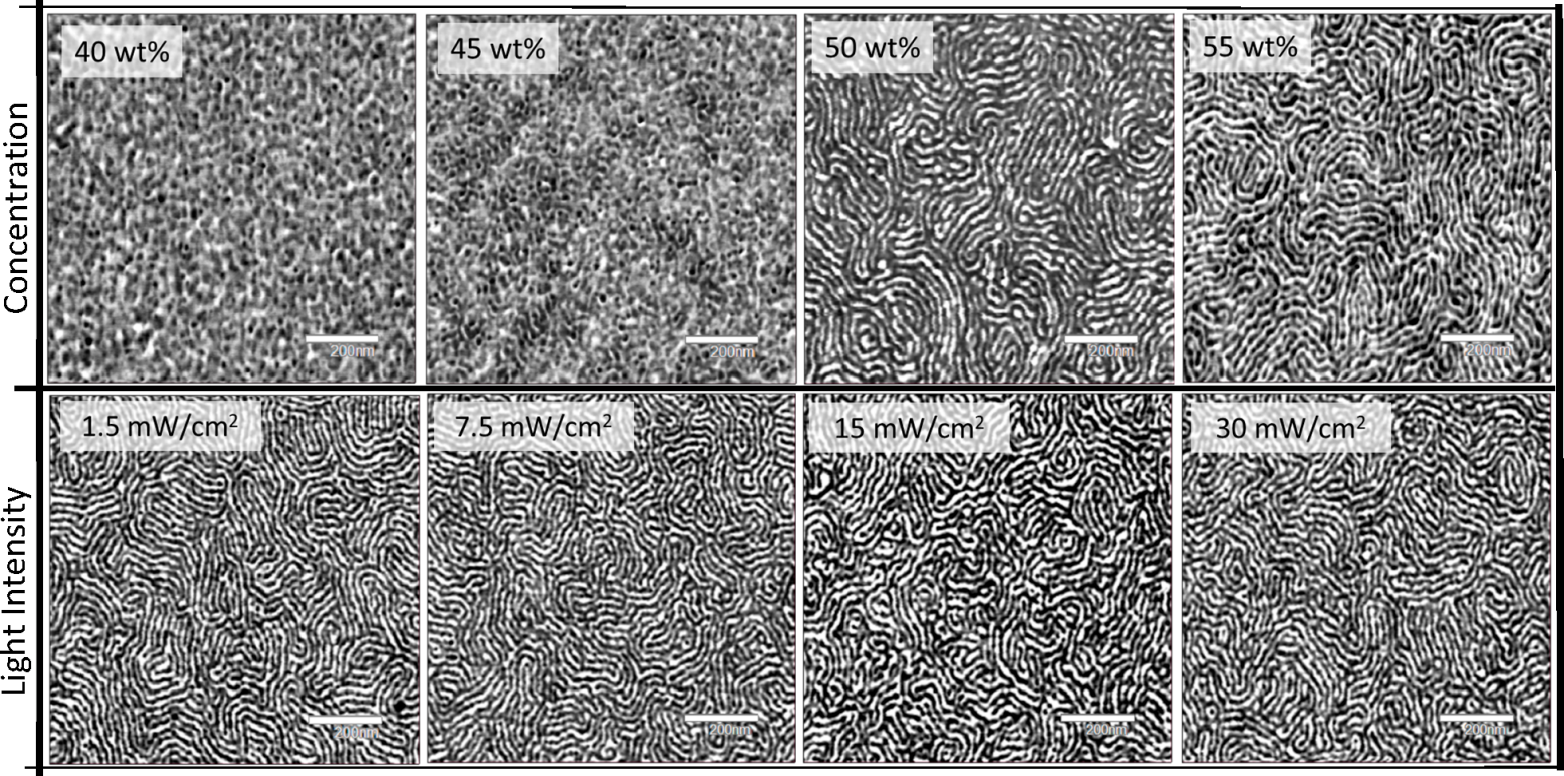
AFM phase images showing effects of diblock copolymer concentration (top row) and photopolymerization light intensity (bottom row) on the nanophase-separated morphology of epoxy networks. Top row: concentration was varied from 40 to 55 wt %, and photopolymerization was conducted by exposing to 405 nm LED at 15 mW/cm^2^ for 5 min. Bottom row: formulations containing 50 wt % diblock were photopolymerized using a 405 nm LED at various light intensities and holding the total light dose constant (4500 mJ/cm^2^). All samples were thermally annealed at 115 °C for 1 h after photocuring. Image dimensions are 1 × 1 μm^2^, and the scale bar represents 200 nm. Phase angles for all images were normalized to a difference of ±15°.

The evidence of controlled immiscibility between components after network formation indicated that the cocontinuous lamellar structure may be thermodynamically driven through self-assembly of the diblock copolymer before photopolymerization. To investigate compositional effects on nanostructure ordering in the cured state, formulations closer in composition to 50 wt % diblock were examined. AFM phase images as a function of the diblock copolymer concentration are shown in Figure 7 (top row). Decreasing copolymer concentration from 50 to 45 wt % considerably reduced evidence of phase co-continuity. This trend in decreasing phase order was continued by decreasing concentration even further to 40 wt %. Conversely, when the diblock copolymer concentration was increased from 50 to 55 wt %, co-continuity was maintained, and the contrast in phase angle between domains was increased possibly due to even greater immiscibility between the hydrophobic copolymer segments and the epoxy network/hydrophilic segments.

Alternatively, the effects of reaction kinetics on the structure were examined through AFM phase imaging by varying incident light intensity during photopolymerization. Often displayed in LLC systems, phase separation/nanostructure retention can have a strong dependence on polymerization rate due to kinetic entrapment of the mesophase before thermodynamics drives phase separation.^46^ In this way, slower polymerization rates may allow for greater reaction mobility before network gelation and possibly perturb the ordered structure. The polymerization rate was altered by varying light intensity over an order of magnitude while holding the total light dose constant. As shown in Figure 7 (bottom row), the phase images demonstrated very little dependence on light intensity as the network structures appeared quite similar across a broad range of light intensities. Additionally, attenuation of light intensity was studied across the thickness of the material which showed very little structural difference on the surface opposite of incident light exposure (Figure S6). These findings implied that the ordered nanostructure was most likely formed in the resin state, i.e., before photocuring, and is relatively insensitive to polymerization rate, reaction diffusion, and network evolution.

**Figure 8:**
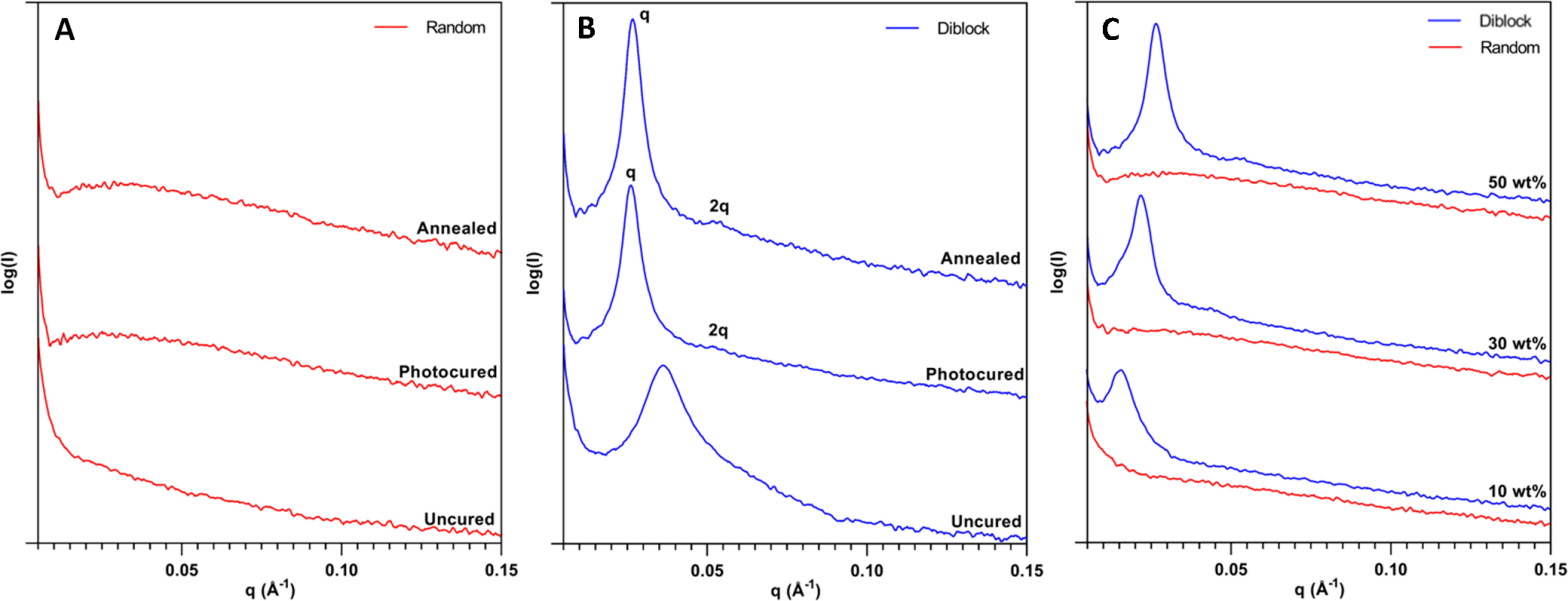
SAXS spectra for (A) 50 wt % random and (B) 50 wt % diblock copolymer-modified epoxy systems at different stages of photopolymer processing. (C) SAXS spectra for photopolymerized and annealed epoxy networks modified with 10, 30, and 50 wt % diblock (blue) or random (red) copolymers.

As a complement to AFM and to further understand the origin of ordered nanophase morphologies, SAXS profiles were obtained before and after polymerization. Figures 8A and 8B show the SAXS spectra for the random and diblock copolymer systems, respectively, at 50 wt % in the epoxy resin. Furthermore, spectra for these systems were recorded at three stages of polymer processing: uncured, photocured, and after thermal annealing. The random copolymer showed no scattering that could be indexed (Figure 8A) which was indicative of an isotropic material containing no ordered heterogeneities on this size scale. After photopolymerization and thermal postcuring, the absence of nanostructure was still apparent and confirms that random functional group placement along the copolymer integrated the additive into the epoxy network with no higher dimensional ordering. Alternatively, the uncured 50 wt % diblock formulation displayed a prominent peak at a *q* value of approximately 0.036 Å^–1^, demonstrating that self-assembly was occurring before photopolymerization (Figure 8B). However, no secondary diffraction peaks were present, suggesting that the amphiphilic copolymer induced a periodic phase morphology but with relatively low degrees of order. After photopolymerization, however, the primary peak narrowed and increased in intensity, showing structure retention and possibly greater fidelity in the cured state. Also, a secondary peak was present at 2*q* (0.054 Å^–1^) which can be indexed to a lamellar structure.^26^ The difference in peak width, intensity, and position between the uncured and cured states may have been due to differences in mobility and phase separation. Before cross-linking the material was a viscous liquid which allowed much greater matrix mobility than after network formation. This greater mobility could have possibly reduced the observed degree of order. Moreover, cross-linking of the semihydrophilic diepoxide monomer with hydroxyl pendant groups on the copolymer could have led to greater phase segregation during the photocuring process (i.e., photoinduced phase separation),^5^ thus producing higher definition in the lamellar phase. Furthermore, this primary peak shift to a smaller *q* value suggested increased lamellae spacing. This change may be attributed to the expansion of the deformable, low-*T*_g_ domain in response to the volumetric shrinkage during polymerization in the adjacent high-*T*_g_ domain. Nonetheless, the primary peak maximum of the cured material corresponded to a lamellae width of 23.5 nm and agreed well with the periodic spacing of approximately 20 nm measured via AFM.^26^ Ultimately, these results confirmed that the amphiphilic behavior of the diblock copolymer induced self-assembly in the epoxy resin matrix, and the ordered structure was maintained through polymerization.

The presence of long-range order on the nanoscale was also examined for the photopolymerized epoxy system at lower concentrations of the diblock and random copolymer (Figure 8C). Relative to the 50 wt % formulation, the primary peak position decreased in *q* value to 0.022 Å^–1^ (28.5 nm) and 0.016 Å^–1^ (39.0 nm) for 30 and 10 wt % concentrations, respectively, while no peaks were observed for the random copolymer formulations. In the 30 wt % system, a reduced secondary peak positioned at 0.044 Å^–1^ (corresponding to a multiple of 2*q*) indicated a reduced long-range order relative to the 50 wt % formulation. However, the presence of only a single peak at a lower *q* value in the SAXS spectrum of the 10 wt % diblock formulation denoted a much less ordered structure. Therefore, these results demonstrated that the PBA copolymer segment became less soluble in the phase containing the epoxy/PHEA domain as the diblock copolymer concentration was increased. These increases led to greater self-assembly and higher degrees of ordered nanostructures in the bulk blend.

**Figure 9:**
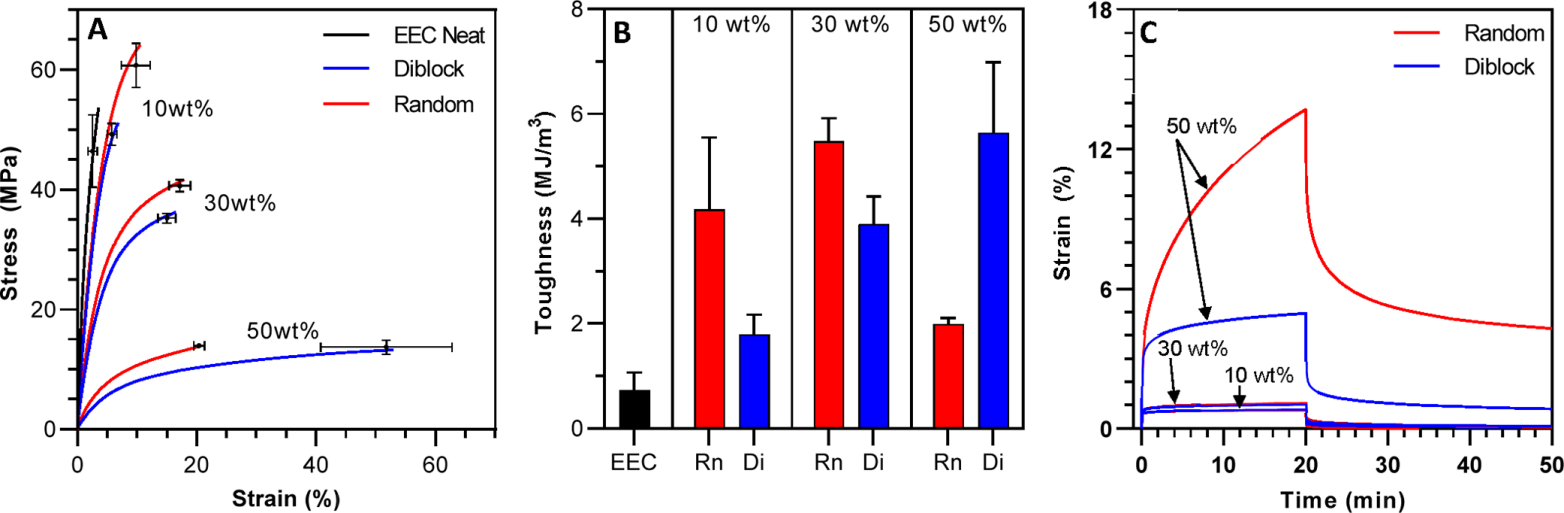
(A) Representative tensile stress–strain curves for epoxy films with 10, 30, and 50 wt % of diblock or random copolymer. Single points and error bars represent the average and standard deviation of three replicates. (B) Tensile toughness for the copolymer-modified epoxy films as represented by the area under the stress–strain curve. (C) Creep strain as a function of time for diblock or random copolymer modified epoxy films at different concentrations. Samples were subjected to a stress of 5 MPa for 20 min and then released. All samples were photopolymerized for 5 min with a 405 nm LED at 15 mW/cm^2^ and subsequently thermally annealed at 115 °C for 1 h.

The presence of ordered nanostructures in these block copolymer blends may lead to significantly modified bulk mechanical properties. Therefore, the tensile properties of these systems were examined as a function of copolymer structure and concentration (Figure 9A–C). Generally, increasing the concentration of copolymer additive, regardless of structure, reduced the tensile modulus accordingly. The reduction in modulus resulted from the increasing content of a rubbery (low *T*_g_) additive which increased network flexibility. Additionally, increases in ultimate strain and decreases in tensile strength were exhibited due to the reduced cross-link density and greater chain mobility relative to the highly cross-linked neat epoxy network. The random copolymer formulation achieved both higher tensile strength and increased elongation when comparing at 10 wt % BCP. The difference in tensile strength and elongation may have been due to greater network integration of the random copolymer whereas the diblock copolymer formed discontinuous low-*T*_g_ domains that simply plasticize the network without providing reinforcement. Increasing concentration to 30 wt %, differences in tensile behavior became less discernible with the diblock system exhibiting similar elongation and slightly reduced tensile strength. Increased continuity of the low-*T*_g_ phase most likely provided greater reinforcement to the bulk material relative to the 10 wt % diblock formulation.

Interestingly, at 50 wt % loading, the diblock copolymer displayed a nearly 3-fold increase in elongation compared to the random configuration, which corresponds to a similar increase in material toughness (Figure 9B). This strain capability increase may be attributed by the cocontinuous lamellar phases that were present in the 50 wt % diblock formulation as shown earlier. With this morphology, after the material reached the point of irreversible deformation (i.e., yield point), the cross-linked network began to fail but was reinforced by interphase adhesion and physical entanglements of poly(butyl acrylate) segments in the low-*T*_g_ phase. The chain entanglements and continuity of this phase likely provided ductility to the material, which enabled greater elongation before material failure while maintaining tensile strength.

Because the diblock copolymer contains a long unreactive segment, it was conceivable that formulations containing this additive may display a reduced creep resistance relative to the isotropic systems. Creep strain as a function of time for the random and diblock copolymer systems is shown in Figure 9C. For these experiments, a constant stress of 5 MPa was applied for 20 min and subsequently released while strain was monitored continuously. At 10 and 30 wt % formulations, very little difference in strain creep was observed, which is most likely due to the predominant mass fraction of the cross-linked network not allowing chain flow under these conditions. However, at 50 wt %, the diblock blend displayed a 2-fold reduction in deformation while under constant pressure compared to the random formulation. Moreover, this composite exhibited considerably increased shape recovery after the load was removed. The increased creep resistance for the diblock system at this concentration is most likely due to the ordered co-continuity of phases. Under fixed stress during these creep studies, the low-*T*_g_ domain allowed some chain mobility resulting in initial deformation. However, the cross-linked high-*T*_g_ domain within the diblock blend limits continued deformation. Thus, the greater local cross-link density imparted by nanostructure provided greater rigidity and resisted deformation more effectively relative to the isotropic system. Owing to the ordered phase-separated structure imparted by the diblock copolymer, this blend showed a nearly 300% increase in toughness combined with a greater than 400% greater creep resistance.

## 4 Conclusion

In this study, block copolymer architecture and concentration were used to induce and control the nanophase separation and thermomechanical properties in photopolymerized epoxy networks. Diblock and random copolymers composed of butyl acrylate and hydroxyethyl acrylate were synthesized by using photoiniferter polymerization. This polymerization method enabled precise control of the pendant group placement, molecular weight, and polydispersity. The two copolymers were nearly identical in composition and MW, enabling direct comparisons between the copolymer configurations. Copolymers were combined with a cross-linking epoxy resin which allowed for covalent connectivity between copolymer and the epoxy network during cationic photopolymerization. The amphiphilic diblock copolymer induced self-assembly of the photocurable system, which significantly increased uncured resin viscosity. The self-assembly led to an increase in the polymerization rate and a reduction in the polymerization gel point at all concentrations examined. Thermomechanical analysis of cured films showed that dual phases were retained through the photocuring process, whereas the random copolymer induced single phase structure. Examination of surface morphology using AFM illustrated that 50 wt % diblock copolymer induced an ordered lamellar structure with nanoscale domain spacing. X-ray scattering confirmed long-range order and demonstrated that ordered lamellar phases were established in the resin phase. Finally, the impact of the controlled nanophase-separated structure on bulk mechanical properties was examined through tensile testing. The lamellar structure exhibited a more than 4-fold decrease in creep deformation, while maintaining tensile strength and doubling ultimate elongation relative to the random, isotropic system. This work shows that phase separation in photopolymers can be controlled using amphiphilic block copolymers to direct the organization of the resin matrix. The combination of ordered high- and low-modulus domains with covalent connectivity between phases may be leveraged to produce materials with greater mechanical resiliency.
