# Supplementary material for: Effect of Block Copolymer Self-Assembly on Phase Separation in Photopolymerizable Epoxy Blends
Source: Macromolecules. 2024 May 15;57(10):4717–28. doi: 10.1021/acs.macromol.4c00192 (PMC11140735; doi:10.1021/acs.macromol.4c00192)
Supplement: Supplementary file 1 — ma4c00192_si_001.pdf [file ma4c00192_si_001.pdf]

Supplementary Information: Effect of Block Copolymer Self-Assembly on Phase Separation in Photopolymerizable Epoxy Blends

Tanner L. Grover[a] and C. Allan Guymon[a,*]
[a]Department of Chemical and Biochemical Engineering, University of Iowa, 4133 Seamans Center, Iowa City, IA 52242, USA
*Corresponding Author: allan-guymon@uiowa.edu

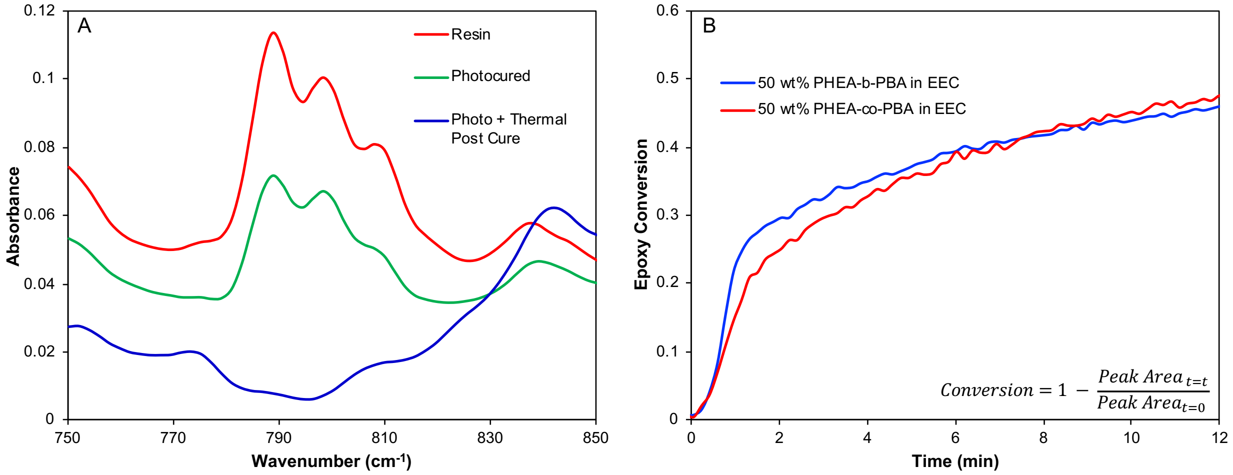

Figure S1 A-B. (A) Fourier Transform Infrared (FTIR) spectra of the oxirane bands (C-O stretch, 775-815 cm^{-1}) for 50 wt% PHEA-*b*-PBA in EEC during the curing process, e.g., uncured resin, after photopolymerization, and thermal post-curing. (B) FTIR real-time conversion of epoxy functional groups as a function of time during light irradiation.

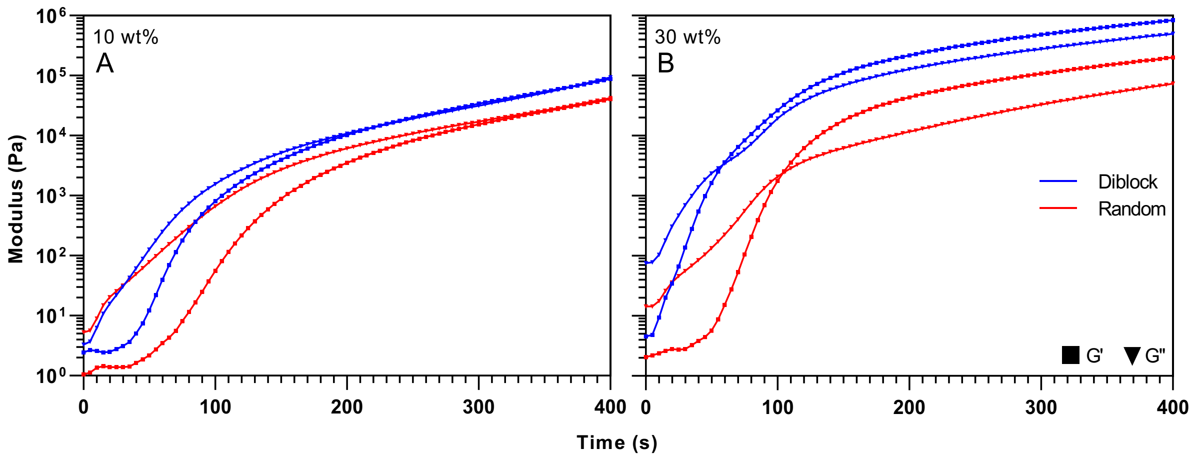

Figure S2 A-B. Storage (G') and loss modulus (G'') as a function of time for blend formulations during photocuring containing (A) 10 and (B) 30 wt% diblock or random copolymer in EEC. The intersection of storage and loss modulus is considered the gel point of the polymerization in this work.

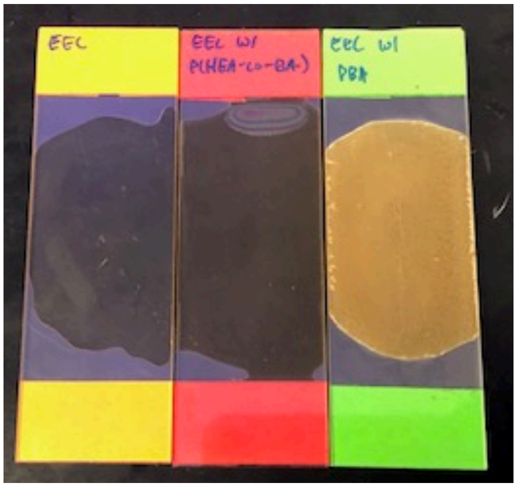

Figure S3. Images of photocured thin films. From left to right: EEC only, EEC containing 20 wt% PHEA-*co*-PBA, EEC containing 20 wt% PBA homopolymer (M_n = 25 kDa).

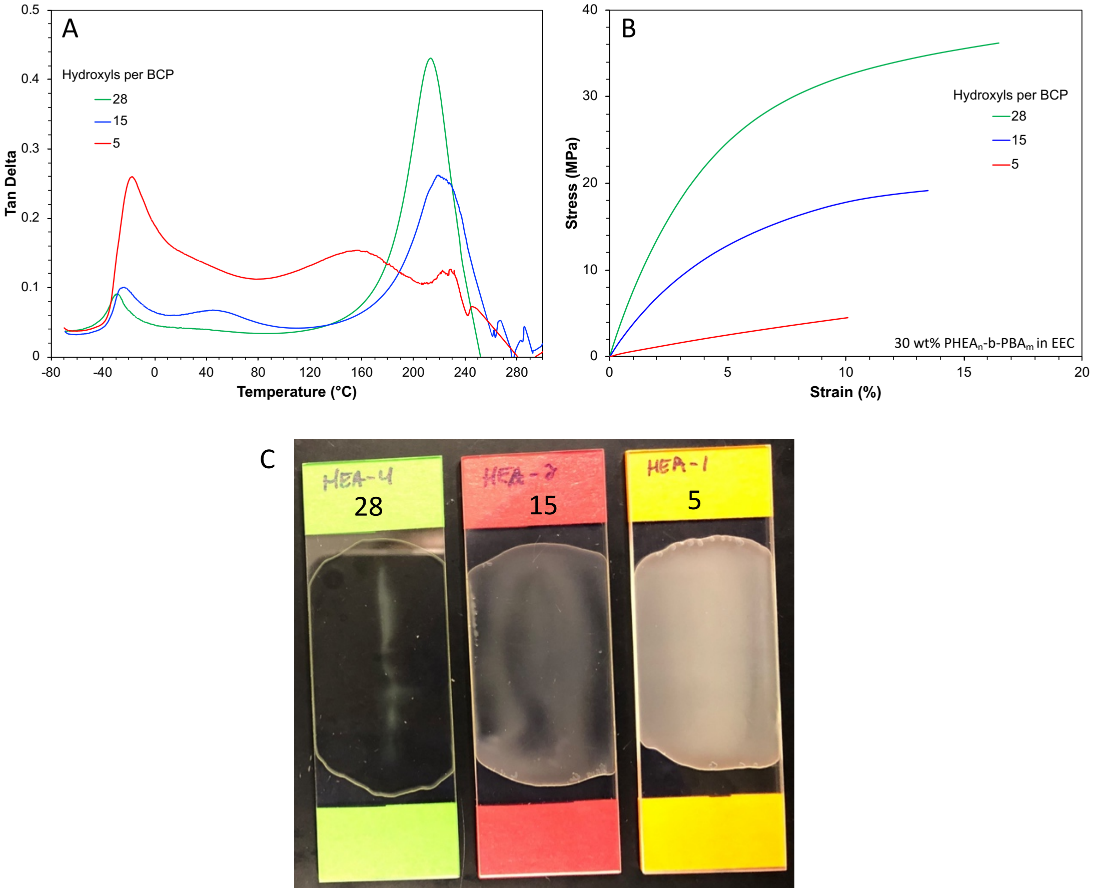

Figure S4 A-C. (A) Tan delta behavior for photopolymerized blends of EEC containing 30 wt% PHEA-*b*-PBA (M_n = 25 – 27 kDa) with varying hydroxyl content. Block copolymer MW was held constant while varying PHEA/PBA segment mass fraction. (B) Corresponding stress-strain behavior for BCP/epoxy blends. (C) Digital image showing increasing opacity of the photocured blends with decreasing hydroxyl content on BCP.

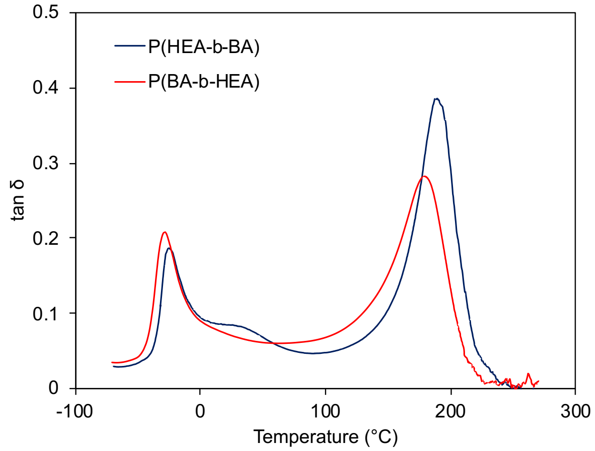

Figure S5. Tan δ as a function of temperature for photopolymerized blends containing 50 wt% PHEA-*b*-PBA or PBA-*b*-PHEA in EEC. The reduced high temperature maximum for P(BA-*b*-HEA) relative to P(HEA-*b*-BA) showed that residual BA pendants were most likely incorporated along the PHEA segment, thus reducing crosslink density in this phase.

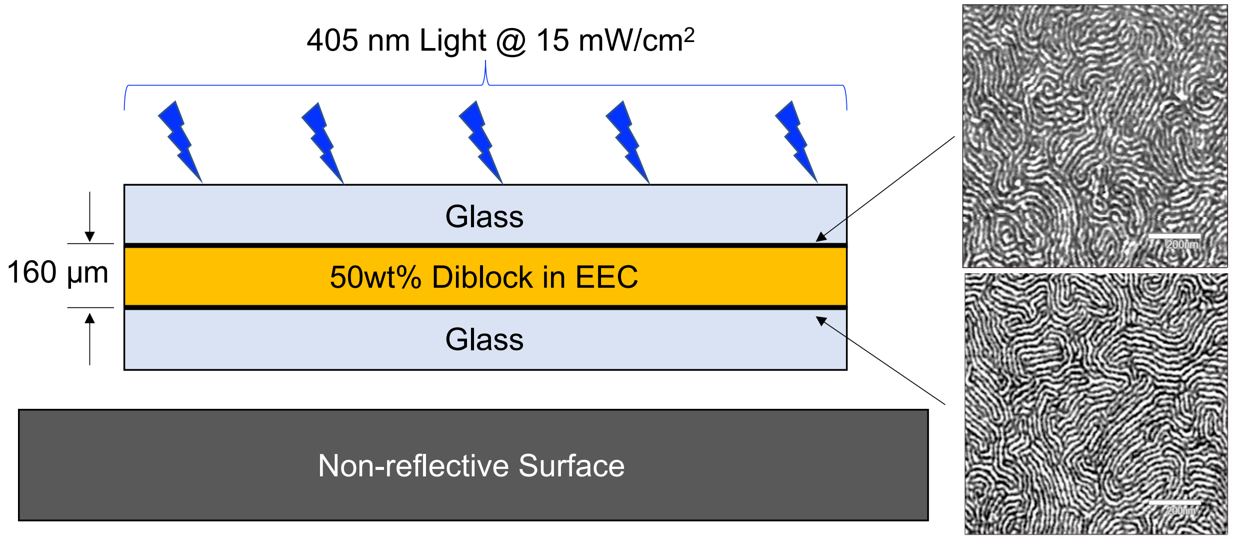

Figure S6. Photocuring scheme for testing light attenuation effects on phase separated structure across film thickness. AFM was conducted on surfaces closest and furthest from light irradiation.
